# Parthenolide regulates microglial and astrocyte function in primary cultures from ALS mice and has neuroprotective effects on primary motor neurons

**DOI:** 10.1371/journal.pone.0319866

**Published:** 2025-03-18

**Authors:** Nadine Thau-Habermann, Thomas Gschwendtberger, Colin Bodemer, Susanne Petri

**Affiliations:** 1 Department of Neurology, Hannover Medical School, Hannover, Germany; 2 Center for Systems Neuroscience (ZSN), Hannover, Germany; University of Rijeka Faculty of Medicine: Sveuciliste u Rijeci Medicinski fakultet, CROATIA

## Abstract

Over the last twenty years, the role of microgliosis and astrocytosis in the pathophysiology of neurodegenerative diseases has increasingly been recognized. Dysregulation of microglial and astrocyte properties and function has been described also in the fatal degenerative motor neuron disease amyotrophic lateral sclerosis (ALS). Microglia cells, the immune cells of the nervous system, can either have an immunonegative neurotoxic or immunopositive neuroprotective phenotype. The feverfew plant (*Tanacetum parthenium*) derived compound parthenolide has been found to be capable of interfering with microglial phenotype and properties. Positive treatment effects were shown in animal models of neurodegenerative diseases like Alzheimer’s disease and Parkinson’s disease. Now we were able to show that PTL has a modulating effect on primary mouse microglia cells, both wild type and SOD1, causing them to adopt a more neuroprotective potential. Furthermore, we were able to show that PTL, through its positive effect on microglia, also has an indirect positive impact on motor neurons, although PTL itself has no direct effect on these primary motor neurons. The results of our study give reason to consider PTL as a drug candidate for ALS.

## Introduction

Amyotrophic Lateral Sclerosis (ALS) is the most common adult onset motor neuron disease. Due to degeneration of motor neurons in the primary motor cortex, brainstem and spinal cord, patients develop progressive muscle weakness and atrophy and ultimately die within 2–5 years, often from respiratory failure. While the majority of ALS cases are sporadic (sALS), up to 10% of patients suffer from familial ALS (fALS). These two forms of ALS are clinically indistinguishable and share many common pathogenic pathways [1]. Mutations in the superoxide dismutase 1 (SOD1, first identified 30 years ago, are responsible for up to 20% of familial cases [2]. Advances in genetics have significantly expanded the spectrum to more than 40 known ALS-related gene mutations [3] but mice overexpressing mutant SOD1 remain the best characterized animal model to date, closely resembling the human ALS phenotype [4].

Microglia cells are immune cells of the nervous system and influence the development and the maintenance of the neural environment. Depending on environmental stimuli, they can change their reactive state from a proinflammatory so-called M1 phenotype to a neuroprotective M2 phenotype or the other way around [5,6]. In ALS, reactive microglia cells contribute to disease onset and progression [7,8] and therapies modifying the reactive state and intrinsic microglial functions represent a promising novel approach. It has been shown that mutant SOD1 microglia cells are more neurotoxic relative to wild type microglia cells [8,9]. Primary microglia cells prepared from lumbar spinal cord of the mutant SOD1 mouse model revealed diminished mRNA expression of M2 markers and enhanced mRNA levels of M1 markers [10]. Not only a shift towards factors promoting M1 phenotype (tumor necrosis factor α (TNF-α), interleukin 1β (IL-1*β*), IL-18, IL-6, and endothelial nitric oxide synthase (iNOS)) can be observed in ALS mouse models and patients but also the respective signaling pathways (nuclear factor kappa-light-chain-enhancer of activated B cells (NF-𝜅B), p38 mitogen-activated protein kinase (p38MAPK), extracellular signal-regulated kinases 1 and 2 (ERK1/2), and Signal transducer and activator of transcription 3 (STAT3)) seem to be affected in ALS [11–19]. It has furthermore been demonstrated that IL-4 gene therapy had beneficial effects in mutant SOD1 mice by changing the microglial phenotype [20].

Astrocytes, as one of the most abundant cell populations in the central nervous system (CNS), are essential for CNS functions in health and disease. They interact with both neural and non-neural cells, such as neurons, oligodendrocytes, or microglia, and, in healthy CNS tissue, maintain homeostasis of extracellular fluids, ions, and transmitters, provide glucose metabolites as energy substrates for neurons, and play an essential role in synapse development and plasticity [21–23]. The role of astrocytes in ALS pathogenesis is very diverse and complex. A dynamic interplay between loss of function mechanisms (such as decrease in lactate levels and impairment of lactate efflux, lower secretion of antioxidants, or lack of microRNA-containing vesicles [24–27]), and gain of toxic function (e.g., release of cytokines such as TNF-α, IL-6 and IL-1β as well as, nitric oxide (NO), ROS and upregulation of inflammatory NF-kb pathway [28–32]) must be assumed. Meyer and colleagues have shown that astrocytes from post-mortem tissue of sALS or fALS are toxic to healthy motor neurons in cell culture and that astrocytes are involved in different mechanisms during ALS progression [33,34], via loss of homeostatic functions or gain of toxic functions, whereas Smethurst and colleagues have shown that healthy astrocytes protect motor neurons [34]. Reactive microglia cells responding to lipopolysaccharide (LPS) secreted certain substances, such as IL-1α or TNF-α and thus triggered astrocyte activation, which in turn had a toxic effect on neurons [35,36].

Parthenolide (PTL), a sesquiterpene lactone which occurs naturally in the plant feverfew (*Tanacetum parthenium*), has first been explored as a potential treatment for migraine [37–41], glioma and peripheral nerve damage [42–44]. Positive treatment effects with PTL or PTL derivatives were also shown in cellular and animal models of neurodegenerative diseases like Alzheimer’s disease [45,46] and Parkinson’s disease [47,48]. Moreover, PTL has anti-inflammatory effects and the potential to reduce LPS induced release of TNFα, circulating cytokines and markers of brain inflammation [49–53]. In addition, it protects from oxidative damage by inducing DNA repair via the PI3- kinase-dependent Nrf2/ARE pathway [54] and inhibits LPS-induced NO and iNOS synthesis in primary rat microglia cells, probably mediated by inhibition of LPS-induced phosphorylation of p42/44 MAPK [55].

Mainly focusing on the impact of PTL on a shift of the reactive state of immunonegative/neurotoxic microglia cells towards a neuroprotective phenotype, but also on additional effects on other related ALS pathomechanisms, we have now analysed the effects of PTL in vitro in primary motor neurons, microglia and astrocyte mono- and co-cultures from SOD1G93A-ALS mice. We further explored whether a shift of the reactive microglia state can also have a positive impact on reactive astrocytes and on the survival of motor neurons.

## Materials and methods

### Animal husbandry/breeding and tissue removal

The experiments were in accordance with the German Animal Welfare Legislation and approved by the local Institutional Animal Care and Research Advisory Committee and permitted by the Lower Saxony State Office for Consumer Protection and Food Safety (§4, TierSchG: reference number 42500/1H; Breeding permission: 18/3016). Animals were only used for tissue collection to generate wild type and transgenic primary cell cultures and were not used for experiments (no treatment with anaesthetics or analgesics took place). The animals did not suffer any pain. Since, according to German law, tissue collection is not considered as an animal experiment in the strict sense, there were no exclusion criteria. Tissue removal was documented and reported to the local Institutional Animal Care and Research Advisory Committee in each case. No calculation of the animal sample size was made in advance. The number of animals used depended on the amount of material obtained for cell cultures that could be used for the subsequent analyses. More detailed information can be found in the corresponding sections on microglia cells and motor neurons.

Transgenic male mice over-expressing the human SOD1G93A mutation, strain B6.Cg-Tg(SOD1-G93A)1Gur/J (high copy number, RRID:IMSR_JAX:004435) [4] were purchased from the Jackson Laboratory (Bar Harbor, ME, USA). Mice were housed under controlled conditions in the Central Animal Facility of Hannover Medical School with a room temperature of 22 ± 2°C, humidity of 55 ± 5% and maintained on a 12:12 h light: dark cycle. Mice of the same sex that were not in mating were housed in groups of up to five animals in open caging in Makrolon cages type II (Uno, Zevenaar, Netherlands). The animals were provided with houses and nest building material. It was ensured that the animals had free access to food (Altromin-Zuchtfutter (Altrogge, Lage, Germany)) and water over the whole time. Mice were mated in a 1:1 or 1:2 mating for a few days. Subsequently, females (B6 mice-breeding of the Central Animal Facility of Hannover Medical School) were kept in groups of up to five animals and singled when pregnancy was visible. The pups were taken from the mothers for preperation at 0-3 days of age. Groups of pups were always gender matched. Transgenic progeny were identified by polymerase chain reaction (PCR) amplification of tail DNA. Primer sequences for the SOD1G93A PCR were adopted from Alexander et al. [56]. Primers for conventional PCR were synthesized by MWG (Ebersberg, Germany) as follows: hSOD1 forward CAT CAG CCC TAA TCC ATC TGA, hSOD1 reverse CGC GAC TAA CAA TCA AAG TGA, and for internal control IL-2 forward CTA GGC CAC AGA ATT GAA AGA TCT and IL-2 reverse GTA GGT GGA AAT TCT AGC ATC ATC C.

### Preparation of primary mouse mixed glial cells

For all experiments primary cultures of mixed glial cells were prepared from neonatal mice P1–P3 of wild type and SOD1G93A mice as described (Prajeeth et al 2014). Neonatal mice were prepared from wild type female mice who were mated with transgenic SOD1G93A (B6.Cg-Tg(SOD1 * G93A)1Gur/J) male mice. The pups of 41 litters (on average 6 pups per litter) were decapitated in accordance with the German Animal Welfare Legislation (without use of anesthetics). The brains were freed from meninges and digested enzymatically with 0.1% trypsin (Sigma-Aldrich) and 0.25% DNase (Roche, Mannheim, Germany) and filtered through a 70 µm strain. The single cells were seeded into pre-coated poly-l-lysine-coated (Sigma-Aldrich, Hamburg, Germany) T-75-mm^2^ culture flasks and filled up with medium consisting of Dulbecco’s modified Eagle’s medium (DMEM; Life Technologies Carlsbad, USA), 1% penicillin/streptomycin (Sigma-Aldrich, Hamburg, Germany) and 10% fetal bovine serum (FBS; Biochrom, Berlin, Germany). After one, five and 10 days the medium was completely replaced by fresh medium. During this time cultures were incubated at 37 °C and 5% CO_2_.

### Generation of primary mouse microglia cells and their treatment design

Microglia cells were isolated on day 11 by shaking at 37°C for 15 h at 110 rpm on an orbital shaker (Edmund Bühler, Heching, Germany) and afterwards pooled, plated with 50,000 cells/well in 24-/96-well plate (Nunc Nunclon Surface), 125,000 cells/well in 12-well and 400,000 cells/well in a 6-well plate.

After resting for 24 h, medium was refreshed and cells were stimulated with 100 ng/ml LPS (from *Escherichia coli* 0111:B4, Merck, Darmstadt, Germany) for one hour and treated afterwards with several concentrations of parthenolide (Santa Cruz Biotechnology, Santa Cruz, California, USA) for again 24h.

### Generation of primary mouse astrocytes and their treatment design

For isolation of transgenic astrocytes, remaining microglia and oligodendrocyte progenitor cells were eliminated by cytosine arabinoside (AraC; 100 μM; Sigma-Aldrich) treatment for 24h followed by shaking the flask at 240 rpm for 6 h (Protocol Biolegend https://www.biolegend.com/en-us/protocols/isolation-and-culture-of-primary-mouse-astrocytes). After these 30h medium containing AraC together with shaken-off cells was removed and the astrocytes were washed two times with phosphate-buffered saline (Sigma-Aldrich, Hamburg, Germany). After mild harvesting in Accutase solution (StemPro Accutase, Gibco, Life technologies, Carlsbad, California, USA) astrocytes were plated with a density of 400,000 cells/well into 6-well plates (one animal per set) with a resting time of at least 18h. Medium was removed and replaced by the treated microglia medium for further 24 h (see Fig 4).

**Fig 1:**
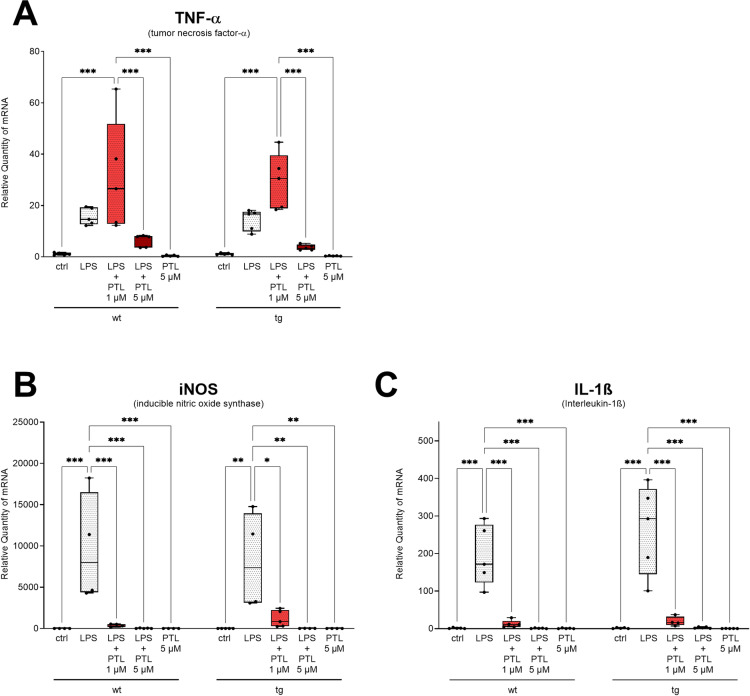
Quantitative analysis of mRNA expression levels of treated wild type (wt) and transgenic (tg) microglia cells compared to untreated microglia cells (ctrl). LPS induced increases in (A) TNF-α, (**B**) iNOS and (C) IL-1β mRNA expression. Treatment with PTL resulted in reversal of the LPS-induced changes of the reactive state of wild type and transgenic SOD1G93A-primary microglia cells by significant downregulation of the mRNA expression levels of inducible nitric oxide synthaseand interleukin-1 beta. 2-ΔΔCt method was used for the quantification of mRNA expression and results were compared by two way ANOVA followed by Tukey´s Multiple Comparison Test ((A) F(9,36) = 10.800; n = 5 (number of independent cell culture preparations); P < 0.001; (B) F = (9,29) = 7.985; n = 4–5 (number of independent cell culture preparations); P < 0.001; (C) F(9,35) = 21.020; n = 4–5 (number of independent cell culture preparations); P < 0.001; the data are illustrated graphically as a box plot from min to max; * p <  0.05; **p <  0.01; ***p < 0.001).

**Fig 2:**
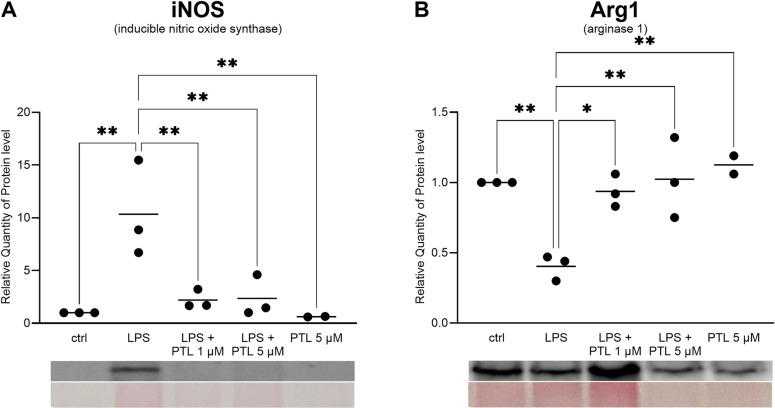
Quantitative analysis of protein levels of iNOS (A) and Arg-1(B) of 400k non/treated transgenic microglia cells via Western Blotting. Values were normalized to whole protein concentration. Results were compared by two-way ANOVA followed by Tukey’s Multiple Comparison Test ((A) F(4,7) = 12.73; n = 3 (number of independent cell culture preparations); P = 0.003; (B) F = (4,7) = 11.03; n = 3 (number of independent cell culture preparations); P = 0.004; the data are illustrated graphically as a box plot from min to max; * p <  0.05; ** **p** <  0.01; ***p < 0.001).

**Fig 3:**
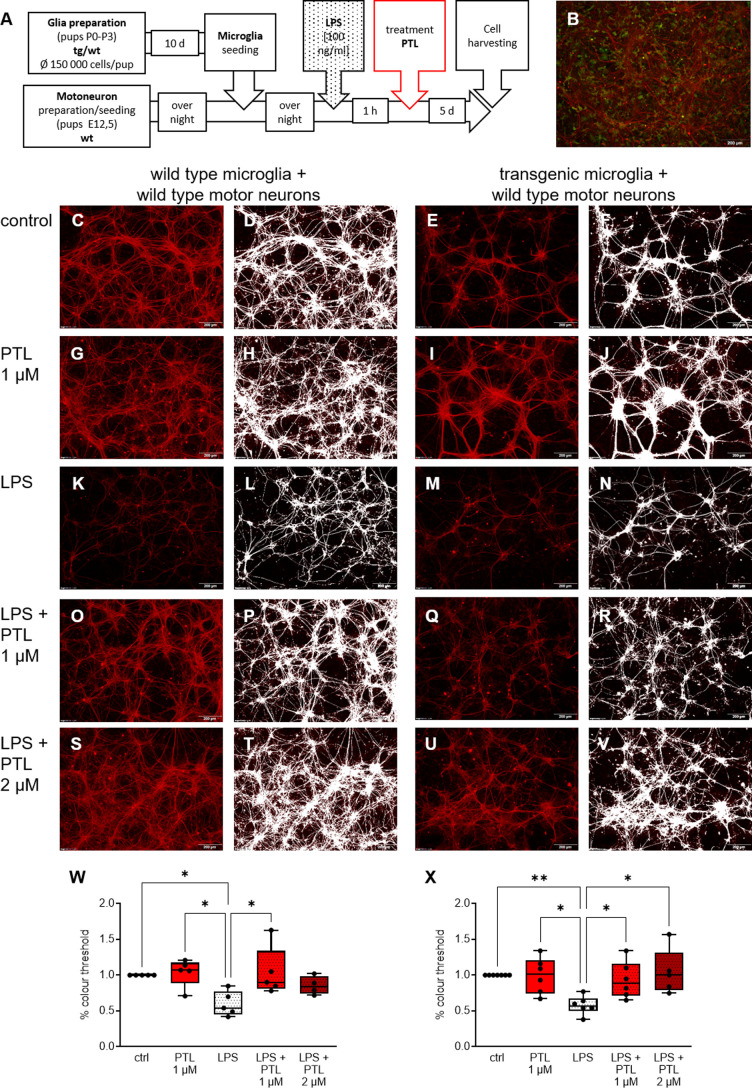
Cell area analysis of treated and untreated motor neurons in co-culture with microglial cells (A) Workflow of glial cell and motor neuron preparation and exposure to LPS and PTL. (**B**) Representative staining of transgenic microglia co-culture with wild type motor neurons stained with SMI32 (red) and Iba1 (green). (**C-U**) Representative series of immunocytochemical stainings of wild type (**W**) and transgenic (**X**) microglia co-culture with wild type motor neurons stained with SMI32 (red) at the respective conditions. Motor neurons are tracked (white) for further analysis. Quantitative analysis of the ICC staining of wild type (**W**) and transgenic (**X**) motor neurons demonstrated that microglial activation by LPS led to a significant reduction in neuronal growth of motor neurons. Parthenolide treatment alone did not result in any change in comparison to the control condition (culture medium). LPS-induced toxicity was reversed both in wild type and transgenic motor neurons by PTL leading to a significant increase in motor neuronal growth compared to the cultures treated with LPS alone. Results were compared by two-way ANOVA followed by Tukey’s Multiple Comparison Test ((**W**) F(4,15) = 4.315; n = 4–5 (number of independent cell culture preparations); P = 0.02; (**X**) F = (4,19) = 5.459; n = 5–7 (number of independent cell culture preparations); P = 0.004; the data are illustrated graphically as a box plot from min to max; * p <  0.05).

**Fig 4.**
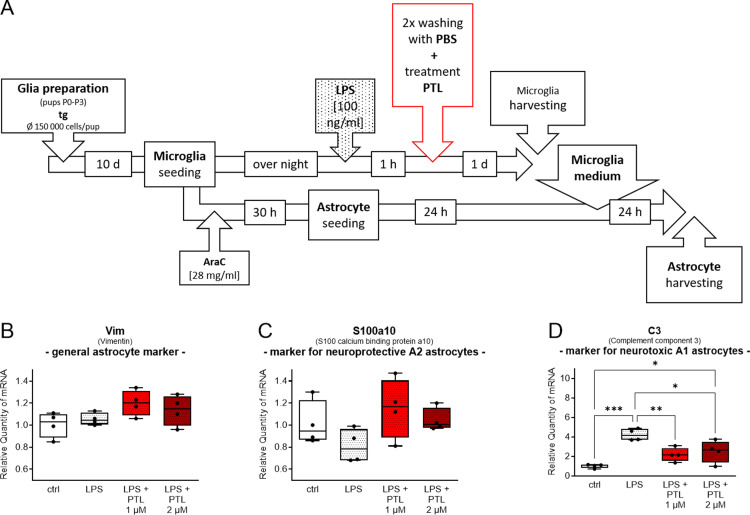
(A) Workflow of microglia cells and astrocyte preparation and subsequent treatment. (**B-D**) Quantitative analysis of qPCR of astrocytes treated with microglial conditioned medium. Induction of toxicity in transgenic astrocytes by conditioned medium of LPS activated microglia was reversed by additional PTL treatment of microglia. 2-ΔΔCt method was used for the quantification of mRNA expression and results were compared by one-way ANOVA followed by Tukey’s Multiple Comparison Test ((B) F(3,12) = 2.425; n = 4; P = 0.1162; (C) F = (3,12) = 2.207; n = 4; P = 0.1401; (D) F(3,12) = 12.680; n = 4; P = 0.0005; the data are illustrated graphically as a box plot from min to max; * p <  0.05; *** **p** <  0.001).

### Preparation of primary mouse motor neurons and co-culture with microglia cells

Wild type C57BL/6J (RRID:IMSR_JAX:000664) primary motor neurons were obtained from 12.5-day-old embryos based on the protocol of Wiese et al. [57]. For the preparation of the embryos, the mothers (19 animals- on average 6 embryos per preparation) were killed by cervical dislocation (without use of anesthetics) in accordance with German Animal Welfare Legislation. Embryos were decapitated and the spinal cords were used for motor neuron harvesting. All primary motor neurons were pooled together in primary motor neuron medium (Neurobasalmedium Gibco, REF 21103-04; 5% Horse Serum Gibco REF2605-088, 2% B27 supplement (50x) Gibco REF17504-044, 20 ng/ml human BDNF PeproTech Cat#450-02 and 20 ng/ml human GDNF PeproTech Cat#450-10) and 50.000 cells per well of a coated PORN-/Laminin (Poly-DL-ornithine-hydrobromide, Sigma P8638, Laminin (natural, mouse) Gibco, REF 23017-15) 8-well chamber (Falcon® 8-well Culture Slide) were seeded for one days. After 24 h half of the medium was replaced by fresh medium and 50,000 microglia cells in fresh primary motor neuron medium were added to each well. After further 24 h incubation, cells were activated by LPS (dissolved in DMSO) for one hour and then treated with increasing concentrations of PTL (1 µ M, 2 µ M, 5 µ M and 10 µ M, dissolved in DMSO). All conditions not containing LPS or PTL-Treatment received fresh medium and the same amount of carrier solution (DMSO).

### Analysis of MTT assay of primary mouse microglia cells treated with PTL

To determine cell toxicity of the used doses of PTL, microglia cells were seeded in 96-well plates and treated with parthenolide for 24 h. Cell viability was determined by using MTT-assay according to the manufactures protocol. After the LPS/PTL-Treatment for 24 h in the 96 well plate, the medium was carefully removed. One hundred µl of fresh primary motor neuron medium was mixed with 10% MTT (3-(4,5-diimethylthiazol-2-yl)-2,5-diphenyltetrazolium bromide) 5 mg/ml in PBS and 110 µl of the mixture was added to each well of the 96-well plate for an incubation of 4h at 37°C. Thereafter, 85 µl of the medium-MTT mixture was removed and 50 µl of DMSO was added for a short incubation of 10 minutes at 37°C, and the absorbance was measured at 570 nm (background absorbance at 630 nm). Results are shown in the supplementary part, Fig 1).

### Functional analysis of primary mouse motor neurons treated with PTL by Multielectrode Arrays (MEA)

We have used MEA to assess neuronal activity of primary murine motor neurons, measuring extracellular voltage changes to determine the firing patterns of individual neurons. These measurements are non-invasive and allow repeated measurements over several days according to the treatment protocol. For this purpose, as described above, primary murine motor neurons were prepared from 12.5-day-old embryos (4 mothers with an average of 6 embryos each) and 100k in 8µl drops in the center of a PORN-Laminin MEA Cytoview 24 well plate (Cat# M384-tMEA-24W) coated with laminin, as recommended by the manufacturer Axion Biosystems. After one hour, the wells were carefully flooded with 500 µl medium and cultured in an incubator at 37°C and 5% CO2. After 2 days, another 500 µl of medium was added. On days 4 and 7, half of the medium was replaced. On day 9, the viability of the neurons was examined by a 5-minute measurement using Maestro Edge pre-equilibrated to 37°C and 5% CO2 with Axis Navigator (version 3.12.2, Axion Biosystems, USA). After the measurement, 960 µ l of medium was carefully removed and replaced with 960 µ l of medium with and without LPS, including the appropriate PTL addition after 1 hour, depending on the treatment. After 24 and 48 hours, another 5-minute measurement was performed. Briefly, after recording spontaneous neuronal activity, all raw data files were analyzed by using Neural Metric Tool (version 4.1.4, Axion Biosystems USA) for quantification. Hereby the following parameters were used: covered electrode threshold 18 kOhms; active Electrode Criterion were defined as 5 spikes/min; spike> 6xStd Deviation of the internal noise level with a post/pre spike duration of 2,16/0,84 ms of each electrode.

### RNA-Analysis/RT-qPCR

According to the manufacturer’s protocol (RNeasy Micro Kit, Qiagen, Venlo, Netherlands), RNA was obtained from the treated microglia cells/astrocytes transcribed into cDNA using QuantiTect Reverse Transcription Kit (Qiagen, Venlo, Netherlands). Expression of typical microglia cell and astrocyte markers were analysed via RT-qPCR. For quantification of mRNA expression levels of genes listed in Table 1 (and in the supplementary Table 1), the SYBR-Green method with Power SYBR® Green Master Mix (Applied Biosystems, Foster City, Californian, USA) was used. Primers were synthesized by MWG (Ebersberg, Germany) and were used at a quantity of 3.5 pmol each. Equal PCR efficiency of all primer pairs was validated by serial cDNA dilutions. cDNA was used in a concentration of 5 ng per reaction corresponding to mRNA. SYBR green quantitative RT-PCR was performed with the StepOnePlus instrument (Applied Biosystems) under the following amplification conditions: 95°C for 10 min, followed by 40 cycles of 95°C for 15 s and 60°C for 60 s. Specificity of PCR products was ensured by melting curve analyses (95°C for 15 s, 60°C for 60 s and 95°C for 15 s). The relative amount of each gene was calculated via the comparative Ct method as previously described by K. Livak (Applied Biosystems User Bulletin #2, 2001). Ct-values were calculated with StepOne-software version 2.1 using a constant cycle threshold of 0.2. The expression of the gene of interest was normalized against the reference (housekeeping) gene in all samples and gene expression was analysed by 2^-∆∆Ct^ method.

**Table 1:** Primers used for real time PCR.

Genes		Primer sequence (5’- > 3’) (forward/reverse)	Product length
Vim PMID: **28099414**	NM_011701.4	AGACCAGAGATGGACAGGTGA	169
		TTGCGCTCCTGAAAAACTGC	
S100a10 ncbi primer-blast	NM_009112.2	ACTAGCCTCATCGTGGTGTGC	116
		ATCATGGTTTCCATGGCGTGC	
C3 PMID: 23637822	XM_011246258.2 NM_009778.3	AGCAGGTCATCAAGTCAGGC	167
		GATGTAGCTGGTGTTGGGCT	
IL1b ncbi primer-blast	NM_008361.4	TCCTTGTGCAAGTGTCTGAAGC	226
		ATGAGTGATACTGCCTGCCTGA	
TNF-α ncbi primer-blast	NM_013693.3 NM_001278601.1	CCAGTGTGGGAAGCTGTCTT	100
		AAGCAAAAGAGGAGGCAACA	
iNOS ncbi primer-blast	NM_001313922.1 NM_001313921.1 NM_010927.4	GGCAAACCCAAGGTCTACGTT	173
		TCGCTCAAGTTCAGCTTGGT	
PPIA ncbi primer-blast	NM_008907.1	TGCACTGCCAAGACTGAATG	85
		CCATGGCTTCCACAATGTTC	
GAPDH ncbi primer-blast	NM_008084.2	GAACATCATCCCTGCATCCA	77
		CCAGTGAGCTTCCCGTTCA	

### Western blot

After 24 h of treatment with LPS/PTL solution in the 6-well plate, the medium was carefully removed, and the microglia cells were washed twice with PBS solution and transferred to a 1.5 ml tube. After a short centrifugation step to pellet the sample, it was dissolved in 50 µl of RIPA buffer and then homogenized using ultrasound. The whole sample was mixed with Laemmli, denatured in a thermocylcer for 15 min at 95°C, then applied to an 8-16% Mini-PROTEAM TGX Precast Protein Gel (Bio-Rad Laboratories, Inc, Hercules, USA) and finally transferred to Nitrocellulose Blotting Membrane (Amersham Protan Premium, GE Healthcare Life Science, Chicago, USA) by standard procedures inclusive Ponceau S (Sigma-Aldrich, Merck, Darmstadt, Germany) staining according the manufactures protocol. Nonspecific binding sites were blocked with 5% nonfat dry milk (Sucofin, Zeven, Germany) in Tris buffered saline (TBS), while antibodies were diluted in 5% BSA buffer (Sigma-Aldrich, Merck, Darmstadt, Germany). The following antibodies were used: Arg-1 (rabbit, 1:1000 Proteintech, Rosemont, IL, USA; RRID:AB_2289842) and iNOS (mouse, 1:500, Cell Signaling, Danvers, Massachusetts, USA; RRID:AB_1078202). Primary antibodies were incubated overnight at 4°C. Detection was performed with, suitable horseradish peroxidase-conjugated secondary antibodies (1:500; R&D System, Minneapolis, MN, USA; RRID:AB_357235 and RRID:AB_357234) followed by chemiluminescent substrate (SuperSignal West Pico PLUS; Thermo Fisher Scientific, Rockford, IL), and signals were detected by Chemolumineszenz Imager (Intas Science Instruments, Gottingen, Germany). To identify the protein size, Spectra™ Multicolor Broad Range Protein Ladder from Thermo Scientific™ (Catalog number: 26634) was used. The quantification was done using Image Studio Lite Ver 5.2 (LI-COR Biosciences, Lincoln, NE, USA). Values were normalized to total protein and normalized to control the respective blot [58–61].

### Immunocytochemistry of co-culture

After the cells (co-culture of primary motor neurons and microglia cells) had been treated with LPS/PTL solution in an 8-well chamber for 24 h, the medium was carefully removed. Freshly prepared ice-cold 4% PFA solution was used to fix the cells for 20 min at room temperature, and cells were washed twice with PBS solution. Subsequently, fixed cells were treated with a blocking solution (2, 5% BSA, 10% goat serum, 0.3% Triton 100X in PBS) for 1 h at room temperature to block non-specific staining. The primary antibodies (Iba1 rb, 1:1000, RRID:AB_ 839504; SMI32 (anti-Neurofilament H Non-Phosphorylated) ms abcam 1:500, RRID:AB_306084) were diluted in blocking solution, added to the cells and incubated overnight at 4 °C. Then the cells were washed again twice with PBS. The secondary antibodies (anti-goat Alexa 488 RRID:AB_143165, anti-rabbit Alexa 555, RRID:AB_2535769, Thermo Fisher Scientific) were diluted in blocking solution and added to the cells to incubate at room temperature for two hours in the dark. After that, the cells were washed twice with PBS and covered with Mowiol (Roth) according to the manufacturer’s instructions.

### Microscopy and image analysis

As previously described [62], for each well at least two typical pictures were photographed at 10x or 20x magnification using an Olympus BX61 microscope equipped with an Olympus DP72 digital camera and the Olympus CellSens Dimension 1.18 program (Olympus, Hamburg, Germany). Neuronal network density of the motor neurons was analysed using the color thresholding method in ImageJ for the intensity of the staining to achieve a better separation of neuronal network growth and background for a more accurate analysis (http://imagej.net/Auto_Threshold#Default, November 2024). Calibration of the images in Image J was ensured before the measurements. The thresholding method Default was selected via Image Adjust Threshold. Using the same parameters for “Hue”, “Saturation” and “Brightness”, the percentage area of the respective images was used for the analysis of the neural network density. The results were normalized in each individual set to control for the respective approach. Image acquisition and analysis were performed in a blinded manner.

### Statistical analyses

All statistical analyses and graphs were performed using GraphPad Prism 10.4.0 (GraphPad Software Inc., San Diego, Californian, USA). Data were expressed as box plots with min to max (all data points shown) and significance level was set as P < 0.05. Results were compared by one-way or two-way ANOVA with Tukey’s post-test.

## Results

### Purity of cultures and toxicity test

At the beginning we performed purity tests of microglia cells via ICC staining and of astrocytes via RT-qPCR analysis and cytotoxicity measurements via MTT assays. Hereby we confirmed high purities for microglia and astrocyte primary cultures (93% and almost 100%, respectively), as shown in S1 and S3 Figs, and, as shown in S2 Fig, no cytotoxicity of used concentrations of PTL treatment was seen by MTT assay.

### No effect of PTL on pure primary mouse motor neuron cultures

Our MEA measurements showed no significant differences between the individual wells of plated motor neurons before treatment (in S6 Fig as baseline). After 24 hours, only the LPS+PTL 5 µ M group showed a significant decrease in resistance and in firing rate. After 48 hours, this decrease was significantly more pronounced, while all other conditions showed no significant differences between each other. This means that both LPS and PTL alone or in combination had no effect on the neurons up to a concentration of 2 µ M. Only a concentration of 5 µ M PTL after LPS treatment hads a cytotoxic effect.

### PTL-induced intrinsic immunological changes in LPS stimulated microglia cells at RNA level

To evaluate LPS and/or PTL-induced intrinsic immunological changes in microglia cells, wild type and transgenic microglia cells were treated as described above. and tested via RT-qPCR for the specific immunonegative microglia markers TNF-α, iNOS and IL-1β. As demonstrated in Fig 1, PTL reversed LPS-induced changes of the reactive state of wildtype and mutant SOD1G93A-primary microglia cells as revealed by significant changes in mRNA expression levels iNOS (B) and IL-1β (C). For TNF-α, no significant change could be observed with the low concentration of PTL after LPS treatment. However, using the higher concentration of PTL (5 µ M) resulted in restoration of the initial level after LPS and subsequent PTL treatment, as shown in S5 Fig.

### PTL-induced intrinsic changes in LPS stimulated microglia cells at protein level

Further analysis of protein expression of iNOS and its opponent Arg-1 was carried out using Western blotting of transgenic LPS and PTL treated microglia cells. Thereby we found that LPS led to a significant increase of iNOS protein expression which was brought back to the initial level by additional PTL treatment. In contrast, Arg-1 protein levels significantly decreased upon LPS activation and increased again with additional PTL treatment as shown in Fig 2 (B).

### PTL indirectly affects motor neurons in co-culture via microglia cells

We now aimed to evaluate if these positive effects of PTL treatment on microglia cells also had a positive effect on motor neurons in co-culture. Hereby we had to slightly adjust the concentration of PTL treatment, since the higher concentration of PTL (5 µ M) had a negative effect on the survival of motor neurons, as seen by MEA assay (S6 Fig), so that we only doubled the lower concentration from 1 to 2 µ M in the further experimental procedures. Quantitative analysis of motor neuron neural network density by ICC staining showed significant reduction upon microglial activation by LPS which was rescued by PTL at concentrations of 1 and 2 μM (Fig 3 (W) and (X)). While control medium and PTL treatment alone resulted in no significant differences, a significant decrease in neural network density of primary wild type motor neurons was found when LPS was administered to both wild type and transgenic microglia cells. The addition of PTL after LPS treatment reversed this reduction in motor neuron axonal density. Based on the results of the MEA assay in primary motor neurons, it can be concluded that the significant changes of axonal outgrowth in the co-culture with microglia cells and motor neurons are not due to direct effects of LPS/ LPS +  PTL on motor neurons but solely caused by the impact of LPS or LPS +  PTL treatment on microglia cells.

### PTL indirectly affects astrocytes via microglia

In the last part of our study, we investigated the effect of the secretome of LPS/ PTL treated microglia cells on astrocytes via microglia conditioned medium. Primary transgenic microglia cells and astrocytes were obtained according to the scheme in Fig 4A and treated as described above with the only change that after 1h LPS administration the microglia cells were washed twice with PBS before PTL was added. As shown in Fig 4 by the analysis of mRNA expression levels for the three different astrocyte markers, vimentin (Vim), S100a10, and Complement component 3 (C3), there were no differences between groups for the general astrocyte marker vimentin (Vim). Also for S100a10 as a marker of immunopositive astrocytes we could not show differences after exposure to medium of LPS treated microglia cells compared to the control condition, additional treatment of microglia with PTL restored baseline levels to those of control (Fig 4C). C3 as a marker of immunonegative neurotoxic astrocytes showed significant increase after addition of medium of LPS-activated microglia which significantly decreased again when microglia had been treated with PTL after LPS activation (Fig 4D). We further analysed whether control medium or PTL alone induced changes in expression of astrocyte markers when directly applied to the astrocytes in comparison to microglia conditioned medium. As shown in S4 Fig, no significant differences could be found here, so that we assume that the effect is caused solely by effect of PTL on the microglial secretome.

## Discussion

In the present study, we investigated the neuroprotective potential of PTL in wild type and ALS transgenic primary murine cell cultures. We analysed the effect of PTL on microglia cells alone and in co-culture with primary wild type motor neurons. Changes in the expression of pro- and anti-inflammatory mediators were identified both at the mRNA and at protein expression level and growth and survival of motor neurons was increased.

Neuroinflammation is one of the pathological features in the spinal cord of ALS patients both with sporadic and familial ALS. The SOD1G93A transgenic mouse model of ALS has been the first and to date best characterized model of ALS and reflects the impact of non-cell autonomous motor neuron death by activated microglia. The pathophysiological relevance of microglial activation and its impact on the disease course has been characterized most extensively in this model, but it is well known that microglial activation also occurs in human sporadic cases as shown in vivo by radiotracer imaging [63] and in ALS models based on other genetic mutations [64,65]. During the disease course, microglia cells shift towards a toxic phenotype that negatively affects motor neurons [10]. This phenotype can be induced by LPS [66], and results in increased production of proinflammatory cytokines and enzymes such as TNF-α, IL-1β and iNOS [66,67].

This increased gene expression of TNF-α, IL-1β and iNOS triggered by LPS induced microglial activation could also be shown in our primary cultures, both in wild type and transgenic microglia (Fig 1). As described in the introduction, several studies in cell culture and in animal models show that PTL exhibits anti-inflammatory effects and has the potential to reduce LPS-induced microglial release of TNF-α [49–53]. This is in accordance with our results (Fig 1, S5 Fig) showing a similar significant reduction of TNF-α after LPS induction and subsequent PTL administration for both wild type and transgenic microglia cells. In addition, we demonstrated that PTL also significantly reduced the pro-inflammatory cytokine IL-1β after LPS induction, similar to a study in a rat model for neuropathy [68] or in different studies in cell culture [69,70]. LPS-induced increased microglial expression of iNOS significantly reduced upon administration of PTL, which correlates to the results of a study by Fiebich et al. in primary rat microglia cells [55].

The effects of LPS were comparable in both wild type and transgenic microglia regarding induction of proinflammatory mediators. However, transgenic microglia sowed a tendency to require higher concentrations of PTL (5 µ M versus 1 µ M) for similar reduction of iNOS and IL-1β expression.

The enzyme iNOS produces large amounts of nitric oxide (NO), induced in microglia cells in response to inflammatory mediators such as LPS, and is mainly expressed by microglia cells in reactive states in various experimental situations [71,72] and involved in the pathogenesis of neuroinflammatory and neurodegenerative diseases, like stroke, multiple sclerosis, Alzheimer’s disease and ALS [73–76].

Arg-1 plays a central role in regulating the immune response as well, mainly through competition between iNOS and Arg-1 for intracellular arginine, leading to protection of tissues from injury in response to inflammation [72,77]. Quantification of protein expression of iNOS and Arg-1 in transgenic microglia by Western blot (Fig 2) showed an LPS-induced induction of iNOS which was reduced to the normal level by additional PTL treatment. In contrast, Arg-1 level decreased upon LPS activation and increased again with additional PTL treatment reflecting the the competition between iNOS and Arg-1 at the protein level. Our results are consistent with studies in the BV-2 cell line, in rat primary microglia and in N9 microglia cells, reporting that microglia cells exposed to LPS show increased iNOS activation and decreased Arg-1 levels [69,78–81].

Toxic effects of reactive microglia cells on motor neurons in general have previously been demonstrated [82,83]. In the context of ALS, increased neurotoxicity induced by reactive SOD1 microglia cells [8,9] has been reported. It is therefore important to examine a potential drug candidate not only in wild type cultures, but also in corresponding disease models, such as primary cell cultures from transgenic disease models. A switch of the microglial state from an immunonegative to an immunopositive reactive state had a positive effect on SOD1G93A motor neurons in vitro and in vivo [20,84].

We now have shown that activation of both wild type and transgenic microglia has a negative impact on motor neuron survival and that a PTL-induced change of microglial expression of inflammatory mediators results in significant neuroprotection (Fig 3). This is in line with the literature [9,82] showing that microglia cells initially polarized with LPS can be modified towards an immunopositive neuroprotective phenotype inducing significant changes in survival/axonal growth of motor neurons.

MEA assays demonstrated that LPS or LPS + PTL treatment had no effect on the survival and function of neurons. Our co-culture data, on the other hand demonstrate a significant reduction of the neuronal network by LPS administration, while can be preserved by addition PTL. In accordance with the literature [45,85], it appears that LPS administration led to an increased release of TNF-α by the microglia, which had an immunonegative effect on the neurons. Additional PTL treatment after LPS administration significantly reduced this increased release, which had an immunopositive effect on the neurons and thus resulted in a significantly larger neuronal network in the co-culture.

While stimulation with LPS doubled microglial cell viability (as seen by MTT assay), TNF-α increased almost 20-fold. We therefore conclude that the release of TNF-α by microglia cells caused a negative effect on motor neurons, and not just the LPS-induced proliferation of microglia cells alone. As previously described, TNF-α may have led to increased microglial phagocytosis [86]. PTL appears to suppress the release of TNF-α and thus prevent the negative effect of LPS-stimulated microglia cells on motor neurons.

In ALS, not only microglia cells but also other glial cells such as astrocytes have an impact on motor neurons and consequently the course of the disease [87]. Studies on the interactions between microglia cells and astrocytes showed that signals like proinflammatory cytokines such as TNF-α or IL-1β or ROS and NO, originating from microglia or astrocytes, mutually determine fate and function of the respective other cell type [87]. By releasing different signaling molecules, both microglia and astrocytes create an autocrine feedback loop and their bidirectional conversation results in tight reciprocal modulation during CNS injury. Based on their properties, microglia cells may impact reactive astrocyte functions ranging from neuroprotective to neurotoxic [88,89].

Even though the exact mechanism is not yet fully understood, PTL shows inhibitory effects on the cytokine-mediated NF-κB signaling pathway through direct inhibition of IKK-β [90] or indirect inhibitory effects on inflammasome activity via NLRP3 [91], ROS [92] or as an inhibitor of STAT1 and STAT3 phosphorylation[90]. Interaction of PTL with cytokine-mediated activation of signaling is evident in our results in microglial mono-culture and microglia-motor neuron co-culture. It is known from the literature that pro-inflammatory factors such as TNF-α released by microglia cells cause astrocytes to lose some of their original functions and release secretory neurotoxins [93]. A direct correlation between microglia cells and astrocytes and the influence of PTL in this interaction was also recently demonstrated by Noureldeen and colleagues in their study in Wistar rats [94]. This coincides with the indirect effects of PTL on astrocytes obtained by the use of microglia-conditioned medium in our study.

Similar to the study by Liddelow and colleagues [35], we found an induction of astrocyte toxicity by addition of LPS induced microglia-conditioned medium marked by significant increase of the immunonegative neurotoxic marker C3 and decrease of the immunopositive neuroprotective marker S100a10. This effect was reversed when PTL had been added to the microglia cells after LPS induction and the so-conditioned media was added to the astrocytes (Fig 4), resulting in a turnaround with opposite significant expression changes of the two markers [35]. C3 is one of the most characteristic and significantly upregulated genes in immunonegative neurotoxic astrocytes and not expressed in immunopositive neuroprotective astrocytes [35], while the S100 protein family member S100A10 has been identified as specific marker of neuroprotective astrocytes and is essential for cell proliferation, membrane repair and inhibition of cell apoptosis [95,96]. Zamanian and colleagues also detected an increase in C3 in reactive astrocytes after LPS-triggered neuroinflammation in mice [97].

To summarize, we could show beneficial effects of PTL on microglia cells alone and in co-culture with primary wild type motor neurons and indirect effects on astrocytes. Changes in microglial state and function were seen both at the mRNA expression level and at the protein level, which resulted in subsequent improvements in growth and survival of motor neurons. The negative effect of activated microglial secretome on astrocytes, as previously described by Liddelow and colleagues [35,36], was reproduced and its reversal by PTL treatment was demonstrated.

The present study has limitations due to the use of a murine in vitro model derived from embryonic mice at an early stage. It is therefore not fully suitable for modelling the age-related neurodegenerative ALS disease. Even though non cell-autonomous effects of glial cells on motor neurons appears to be a general pathomechanisms in all forms of ALS [98] we only assessed mutant-SOD1 and wild type neurons and glia.

Further studies should add analyses in humanized cellular models and in vivo models to account for different genotypes as the in vitro model used in our study with primary embryonic/ postnatal cells mainly represents mutant SOD1 ALS at an early stage.

While the PTL doses used in these studies were based on previous reports in the literature and our preliminary studies, future studies should also consider dose-response curves to better determine the most appropriate dosage

Our data contribute to the notion that a dialogue exists between the different cell types, especially microglia cells and motor neurons, which has been discussed many times in the literature [99] and shown not only in the field of ALS [7,100], but also other neurogenerative diseases such as Parkinson’s and Alzheimer’s disease [101,102]. Compounds beneficially interacting with the disturbed neuron-glia crosstalk therefore represent interesting drug candidates.

## Conclusion

As there is no cure for ALS so far, it is important to study drugs with potential impact on known disease mechanisms. PTL seems to have effects on factors and pathways involved in ALS onset and progression. Our results show that PTL significantly reverses LPS-induced changes of the reactive state primary microglia cells. While this was not specific for mutant SOD1G93A microglia but also occurred in wild type cells, it resulted in altered mRNA expression levels of TNF-α, iNOS and IL-1β and positively impacted the interplay of the antagonists’ iNOS and Arg-1.

In addition, we found a beneficial effect of PTL-treated microglial cells in co-culture with primary motor neurons, resulting in significant reversal of LPS-induced motor neuron degeneration. Last, we could confirm that reactive microglia cells also modify the reactive status of astrocytes and that treatment of microglia with PTL after LPS-activation induces a neuroprotective phenotype in astrocytes. These results ultimately point to direct or indirect interaction of the three cell types.

Due to this interaction of microglia cells, astrocytes and motor neurons, it is important to precisely characterize the impact of therapeutic candidates on the respective cell types and their crosstalk. In the present study, we have shown for the first time modulation of microglial function by PTL resulting in both beneficial modification of the astrocyte phenotype and in motor neuron protection in the context of the neurodegenerative disease ALS. We therefore see great potential in PTL for successful reversal of dysregulated neuron-glia interaction in ALS.

## Supporting information

S1 Fig Analysis of purity of microglia culture.
For this purpose microglia cells were prepared from neonatal mice P1–P3 of wildtype and transgenic SOD1G93A mice as described (Prajeeth et al 2014). Fifty thousand cells per well of a 24-well plate were seeded for 24h. After resting, medium was refreshed and cells were cultured for further 24h. Freshly prepared ice-cold 4% PFA solution was used to fix the cells for 20 min at room temperature. Afterwards cells were washed twice with PBS solution and subsequently treated with a blocking solution (2, 5% BSA, 10% goat serum, 0.3% Triton 100X in PBS) for 1 h at room temperature to block non-specific staining. The primary antibody Iba-1 (Wako rb, 1:1000) was diluted in blocking solution, added to the cells and incubated overnight at 4 °C. Then the cells were washed again twice with PBS, secondary antibody anti-rabbit Alexa 555 (1:1000, Thermo Fisher Scientific) was diluted in blocking solution and added to the cells to incubate at room temperature for two hours in the dark. After that, the cells were washed twice with PBS and covered with Mowiol (Roth) inclusive DAPI (1:1000 Sigma Aldrich) according to the manufacturer’s instructions. Quantitative analysis of the ICC staining of wild type microglia cells (n = 5 independent cell culture preparations) and a representative staining of wild type microglia cells stained with Iba-1 (red) and DAPI (blue). The microglia culture consisted on average of 93% (86-97%) microglia cells. The data are illustrated graphically as a box plot from min to max.
(TIF)

S2 Fig Analysis of the survival rate of wild type microglia by MTT-Assay. Exposure to LPS and LPS+PTL 1 µ M resulted a higher survival rate compared to the control condition (culture medium). The survival rate of microglia was not affected by PTL alone or by exposure to LPS together with higher concentrations of PTL. Results were compared by one-way ANOVA followed by Tukey’s Multiple Comparison Test (F(5,12) = 10.780; n = 3 (number of independent cell culture preparations); P = 0.0004; the data are illustrated graphically as a box plot from min to max; *  p <  0,05; **p <  0.01; *** p <  0.0001).
(TIF)

S3 Fig Analyse of purity of astrocyte culture. Quantitative analysis of the astrocyte culture of transgenic astrocytes via RT-qPCR. The astrocyte culture consisted of almost 100% astrocytes and a few thousandths of remaining microglial cells, oligodendrocytes and neurons. 2-ΔΔCt method was used for the quantification of mRNA expression and results were compared by one-way ANOVA followed by Tukey’s Multiple Comparison Test (F(6,14) = 556; n = 3 (number of independent cell culture preparations); P < 0.001; the data are illustrated graphically as a box plot from min to max; *** p <  0.0001).
(TIF)

S4 Fig Quantitative analysis of qPCR of astrocytes treated with microglial conditioned medium and control medium with and without PTL-Treatment for all three astrocyte markers. There were no significant differences for the respective markers between control medium and microglia conditioned medium with and without PTL treatment. 2-ΔΔCt method was used for the quantification of mRNA expression and results were compared by one-way ANOVA followed by Tukey’s Multiple Comparison Test ((A) F(3,12) = 1.661; n = 4 (number of independent cell culture preparations); P = 0.2279; (B) F = (3,12) = 1.795; n = 4 (number of independent cell culture preparations); P = 0.2017; (C) F = (3,12) = 2.333; n = 4 (number of independent cell culture preparations); P = 0.1256; the data are illustrated graphically as a box plot from min to max).
(TIF)

S5 Fig Separated quantitative analysis of TNF-α mRNA expression levels of treated wild type (wt) and transgenic (tg) microglia cells compared to untreated microglia cells (ctrl). LPS induced increases in TNF-α mRNA. Treatment with PTL 5 µ M resulted in reversal of the LPS-induced changes of the reactive state of wild type and transgenic SOD1G93A-primary microglia cells by significant downregulation of the mRNA expression levels of TNF-α. 2-ΔΔCt method was used for the quantification of mRNA expression and results were compared by two way ANOVA followed by Tukey´s Multiple Comparison Test (F(7,28) = 52.6; n = 5 (number of independent cell culture preparations); P < 0.001; the data are illustrated graphically as a box plot from min to max; **p <  0.01; ***p < 0.001).
(TIF)

S6 Fig No effect of PTL on pure primary mouse motor neuron cultures. MEA measurements showed that there were no significant differences between the individual wells of plated motor neurons before treatment After 24 and 48 hours, only the LPS+PTL 5 µ M group showed a significant decrease in resistance (A) and in firing rate (B), while all other conditions showed no significant differences between each other. Results were compared by two-way ANOVA followed by Tukey’s Multiple Comparison Test ((A) F(5,54) = 31.39; n = 4 (number of independent cell culture preparations); P < 0.001; (B) F = (5,54) = 7.214; n = 4 (number of independent cell culture preparations); P < 0.001; the data are illustrated graphically as a box plot from min to max; * p <  0.05; ** p <  0.01; ***p < 0.001).
(TIF)

S1 Raw image Supporting information file. (PDF)

S1 Table Primers used for real time PCR. (DOCX)
