# Supplementary figures and images for: Parthenolide regulates microglial and astrocyte function in primary cultures from ALS mice and has neuroprotective effects on primary motor neurons
Source: PLoS One. 2025 Mar 18;20(3):e0319866. doi: 10.1371/journal.pone.0319866 (PMC11918366; doi:10.1371/journal.pone.0319866)

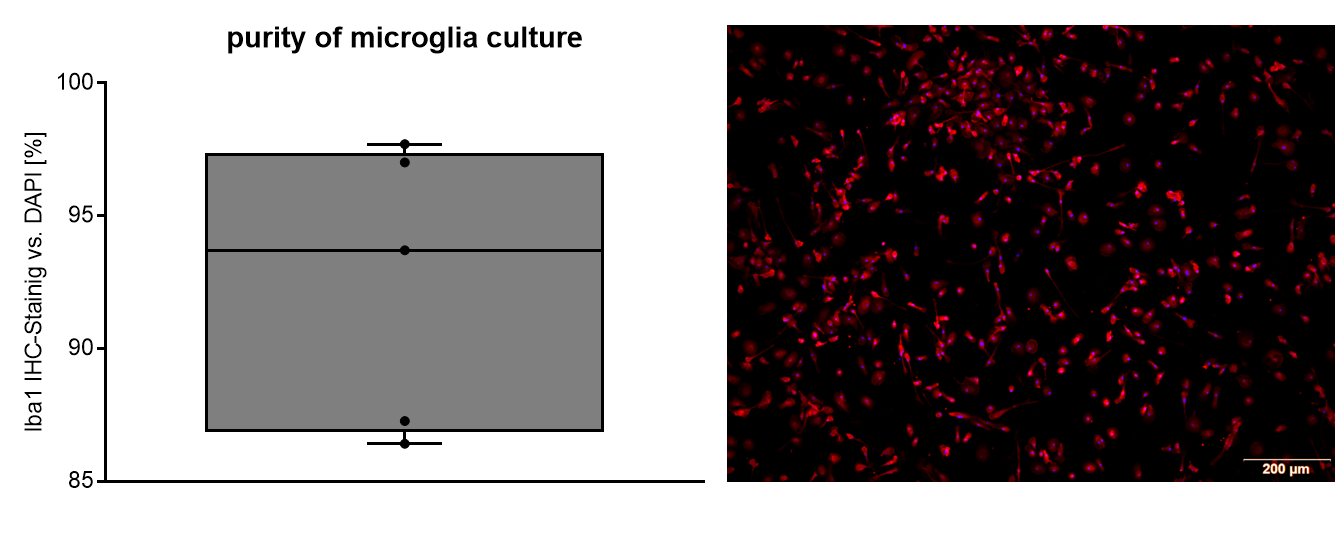

Supplement: S1 Fig — For this purpose microglia cells were prepared from neonatal mice P1–P3 of wildtype and transgenic SOD1G93A mice as described (Prajeeth et al 2014). Fifty thousand cells per well of a 24-well plate were seeded for 24h. After resting, medium was refreshed and cells were cultured for further 24h. Freshly prepared ice-cold 4% PFA solution was used to fix the cells for 20 min at room temperature. Afterwards cells were washed twice with PBS solution and subsequently treated with a blocking solution (2, 5% BSA, 10% goat serum, 0.3% Triton 100X in PBS) for 1 h at room temperature to block non-specific staining. The primary antibody Iba-1 (Wako rb, 1:1000) was diluted in blocking solution, added to the cells and incubated overnight at 4 °C. Then the cells were washed again twice with PBS, secondary antibody anti-rabbit Alexa 555 (1:1000, Thermo Fisher Scientific) was diluted in blocking solution and added to the cells to incubate at room temperature for two hours in the dark. After that, the cells were washed twice with PBS and covered with Mowiol (Roth) inclusive DAPI (1:1000 Sigma Aldrich) according to the manufacturer’s instructions. Quantitative analysis of the ICC staining of wild type microglia cells (n = 5 independent cell culture preparations) and a representative staining of wild type microglia cells stained with Iba-1 (red) and DAPI (blue). The microglia culture consisted on average of 93% (86-97%) microglia cells. The data are illustrated graphically as a box plot from min to max. (TIF) [file pone.0319866.s001.tif]

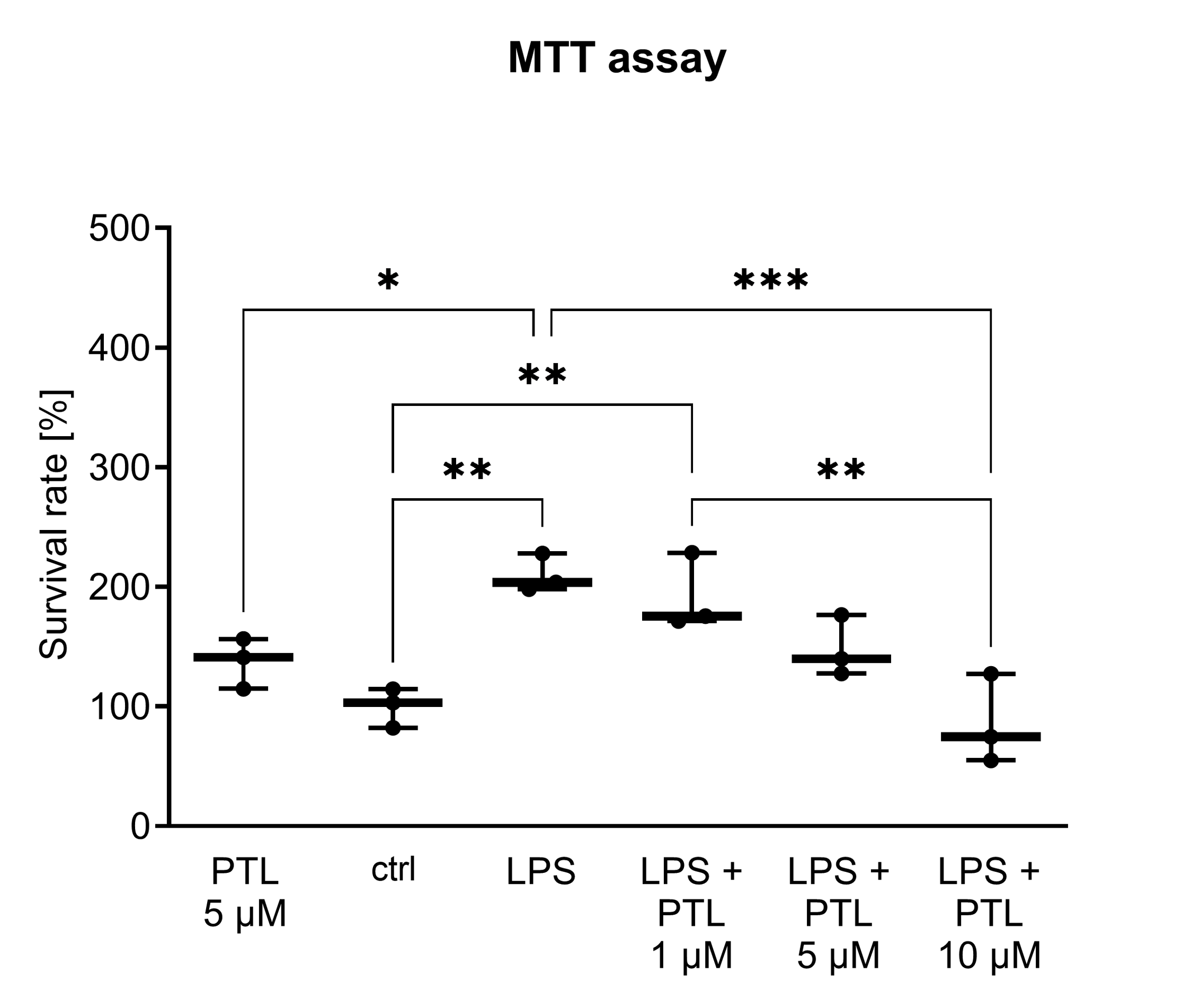

Supplement: S2 Fig — Exposure to LPS and LPS+PTL 1 µ M resulted a higher survival rate compared to the control condition (culture medium). The survival rate of microglia was not affected by PTL alone or by exposure to LPS together with higher concentrations of PTL. Results were compared by one-way ANOVA followed by Tukey’s Multiple Comparison Test (F(5,12) = 10.780; n = 3 (number of independent cell culture preparations); P = 0.0004; the data are illustrated graphically as a box plot from min to max; * p < 0,05; **p < 0.01; *** p < 0.0001). (TIF) [file pone.0319866.s002.tif]

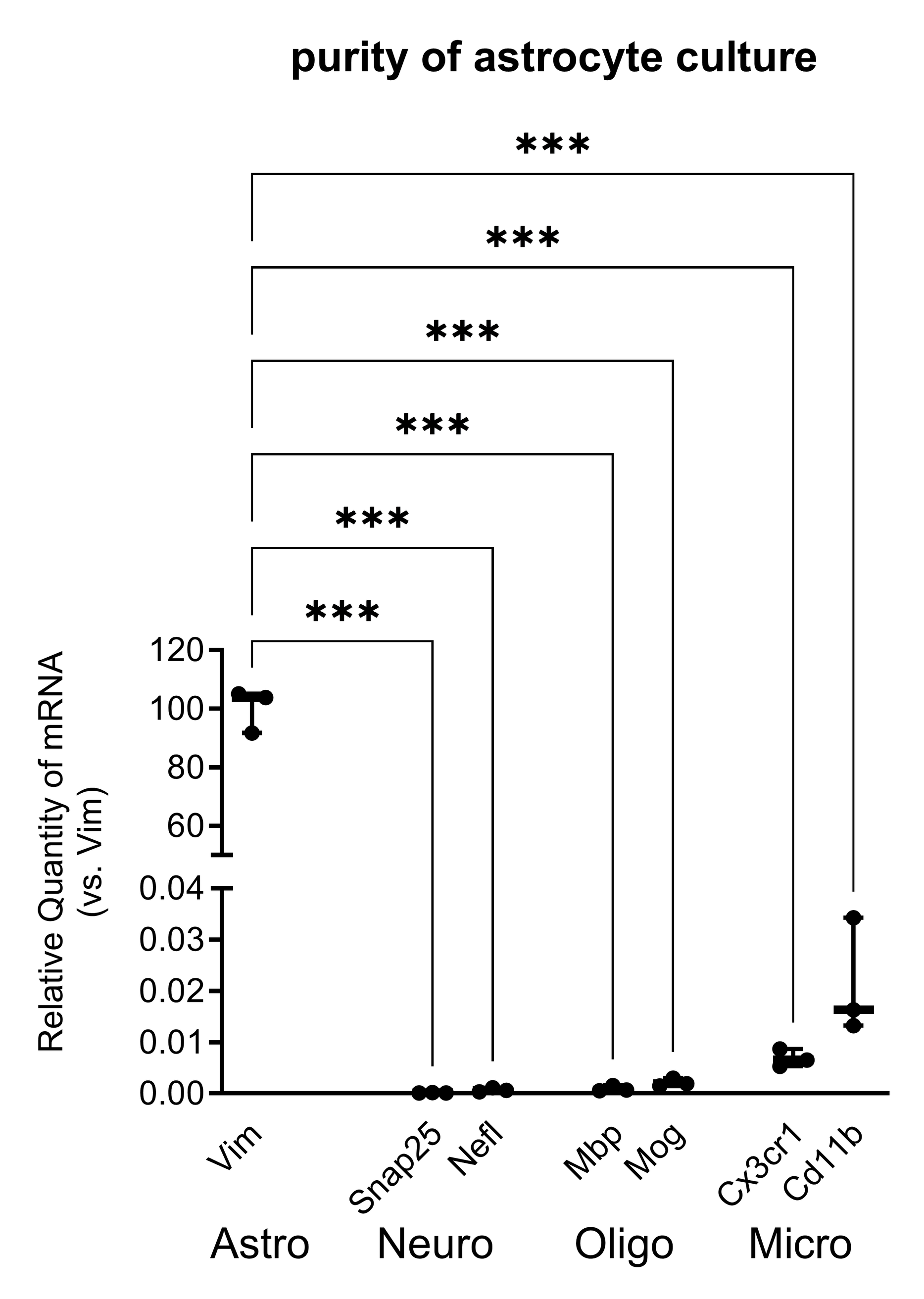

Supplement: S3 Fig — Quantitative analysis of the astrocyte culture of transgenic astrocytes via RT-qPCR. The astrocyte culture consisted of almost 100% astrocytes and a few thousandths of remaining microglial cells, oligodendrocytes and neurons. 2-ΔΔCt method was used for the quantification of mRNA expression and results were compared by one-way ANOVA followed by Tukey’s Multiple Comparison Test (F(6,14) = 556; n = 3 (number of independent cell culture preparations); P < 0.001; the data are illustrated graphically as a box plot from min to max; *** p < 0.0001). (TIF) [file pone.0319866.s003.tif]

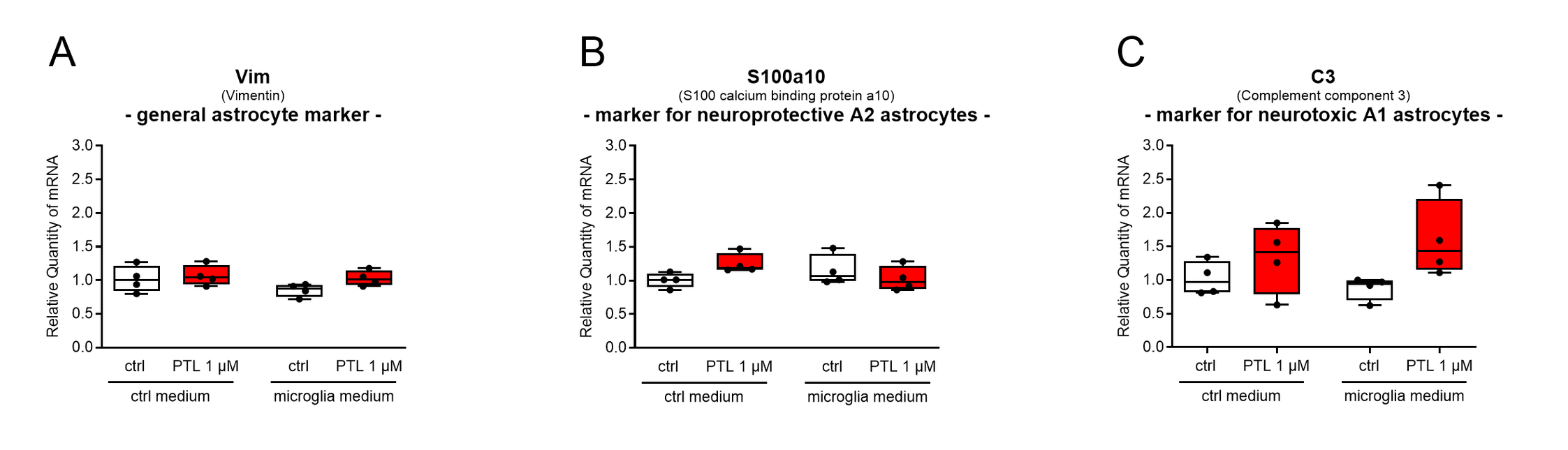

Supplement: S4 Fig — There were no significant differences for the respective markers between control medium and microglia conditioned medium with and without PTL treatment. 2-ΔΔCt method was used for the quantification of mRNA expression and results were compared by one-way ANOVA followed by Tukey’s Multiple Comparison Test ((A) F(3,12) = 1.661; n = 4 (number of independent cell culture preparations); P = 0.2279; (B) F = (3,12) = 1.795; n = 4 (number of independent cell culture preparations); P = 0.2017; (C) F = (3,12) = 2.333; n = 4 (number of independent cell culture preparations); P = 0.1256; the data are illustrated graphically as a box plot from min to max). (TIF) [file pone.0319866.s004.tif]

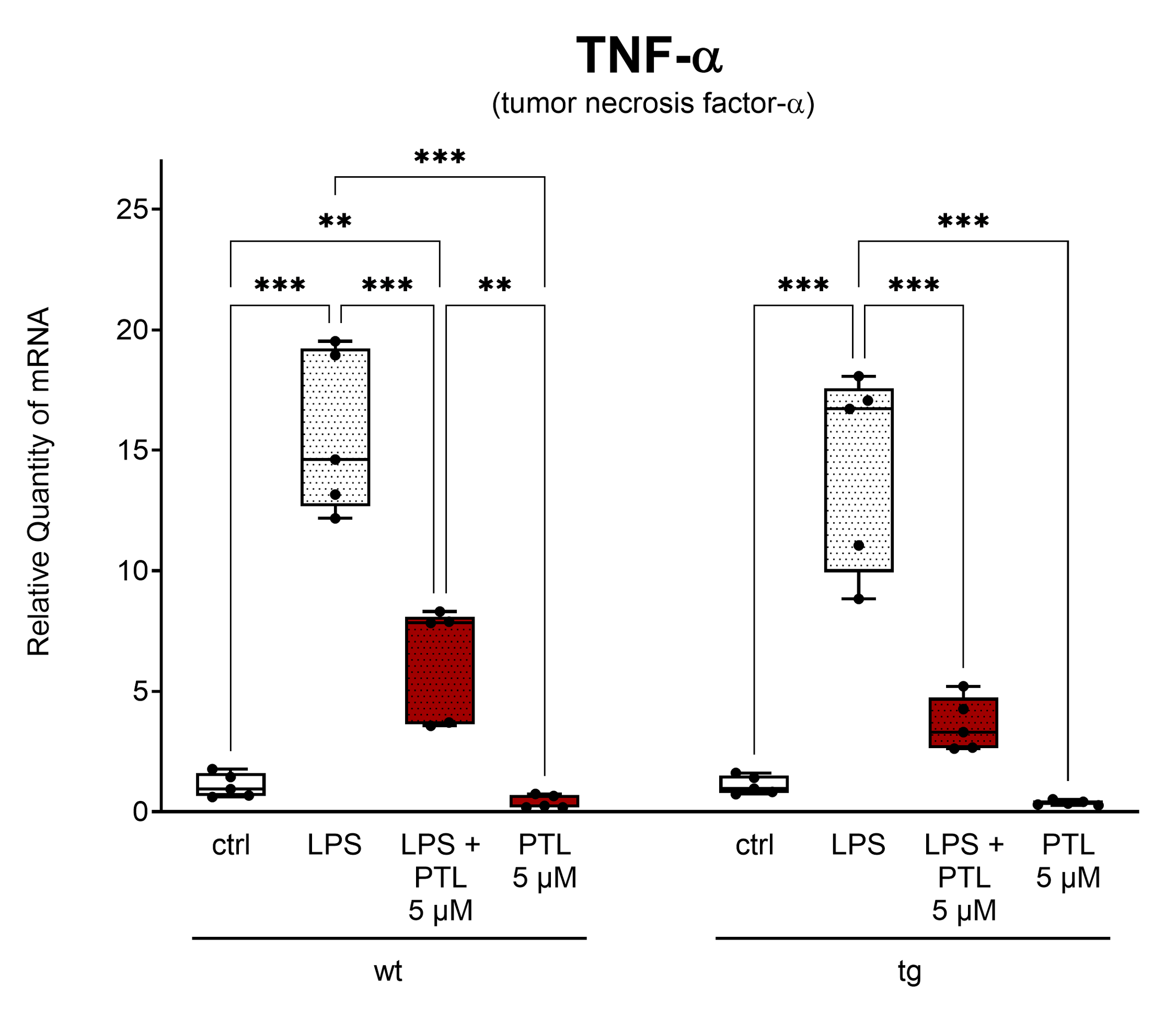

Supplement: S5 Fig — LPS induced increases in TNF-α mRNA. Treatment with PTL 5 µ M resulted in reversal of the LPS-induced changes of the reactive state of wild type and transgenic SOD1G93A-primary microglia cells by significant downregulation of the mRNA expression levels of TNF-α. 2-ΔΔCt method was used for the quantification of mRNA expression and results were compared by two way ANOVA followed by Tukey´s Multiple Comparison Test (F(7,28) = 52.6; n = 5 (number of independent cell culture preparations); P < 0.001; the data are illustrated graphically as a box plot from min to max; **p < 0.01; ***p < 0.001). (TIF) [file pone.0319866.s005.tif]

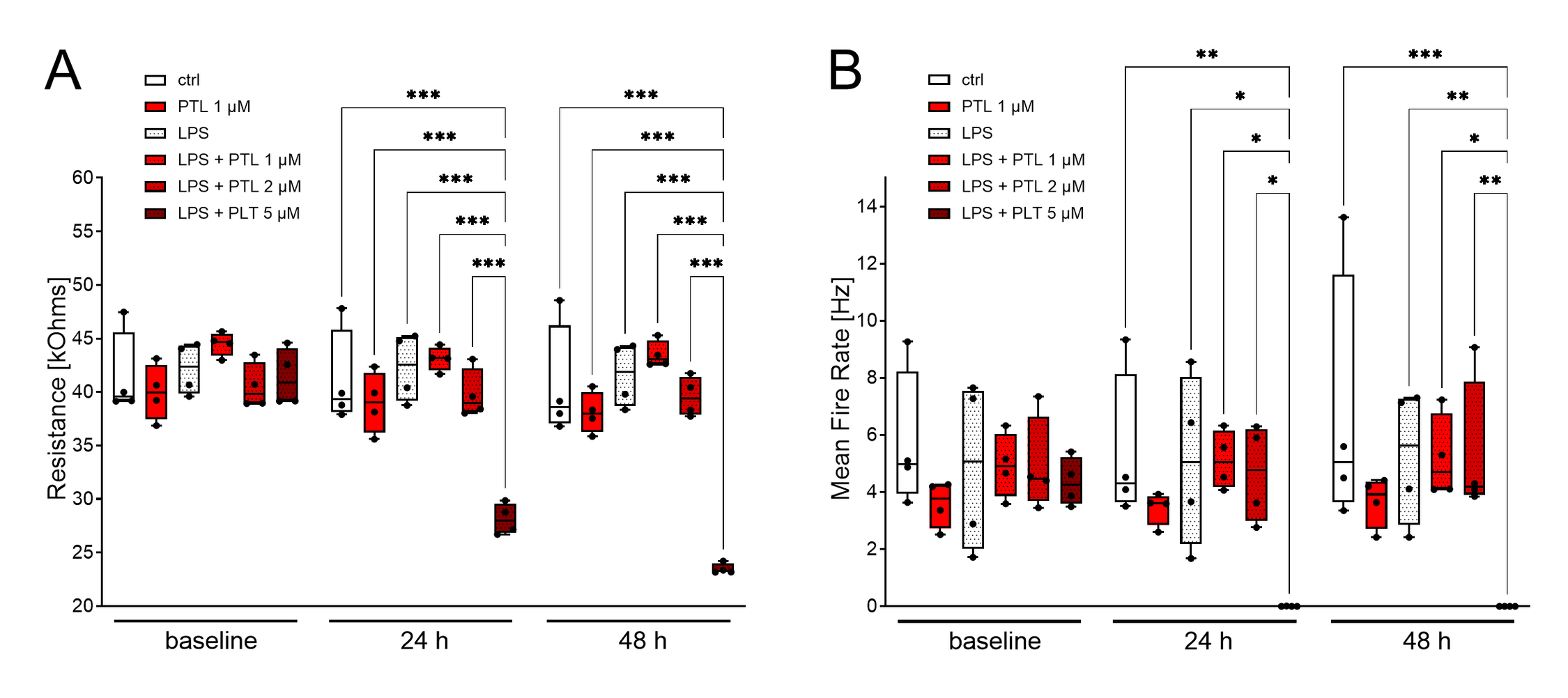

Supplement: S6 Fig — MEA measurements showed that there were no significant differences between the individual wells of plated motor neurons before treatment After 24 and 48 hours, only the LPS+PTL 5 µ M group showed a significant decrease in resistance (A) and in firing rate (B), while all other conditions showed no significant differences between each other. Results were compared by two-way ANOVA followed by Tukey’s Multiple Comparison Test ((A) F(5,54) = 31.39; n = 4 (number of independent cell culture preparations); P < 0.001; (B) F = (5,54) = 7.214; n = 4 (number of independent cell culture preparations); P < 0.001; the data are illustrated graphically as a box plot from min to max; * p < 0.05; ** p < 0.01; ***p < 0.001). (TIF) [file pone.0319866.s006.tif]
