# Supplementary material for: Parthenolide regulates microglial and astrocyte function in primary cultures from ALS mice and has neuroprotective effects on primary motor neurons
Source: PLoS One. 2025 Mar 18;20(3):e0319866. doi: 10.1371/journal.pone.0319866 (PMC11918366; doi:10.1371/journal.pone.0319866)
Supplement: S1 Raw image — (PDF) [file pone.0319866.s007.pdf]

Supporting information file

Parthenolide regulates microglial and astrocyte function in primary cultures from ALS mice and has neuroprotective effects on primary motor neurons

Nadine Thau-Habermann[1*¶], Thomas Gschwendtberger[1,¶], Colin Bodemer[1] and Susanne Petri[1,2]

1 Department of Neurology, Hannover Medical School, Hannover, Germany (Gschwendtberger.Thomas@mh-hannover.de (T.G.); Thau.Nadine@mh-hannover.de (N.T.); Colin Bodemer (C.B.))
2 Center for Systems Neuroscience (ZSN), Hannover, Germany
[¶] These authors contributed equally to this work.

* Corresponding author
E-mail: Thau.Nadine@mh-hannover.de (N.T.)

Abbreviated titel: Positive impact of parthenolide in an ALS in vitro model

Keywords: parthenolide, fewerfew (Tanacetum parthenium), ALS, microglia cells, astrocyte, motor neurons

Raw data of the quantitative analysis of protein levels of iNOS and Arg-1 (Fig 2)

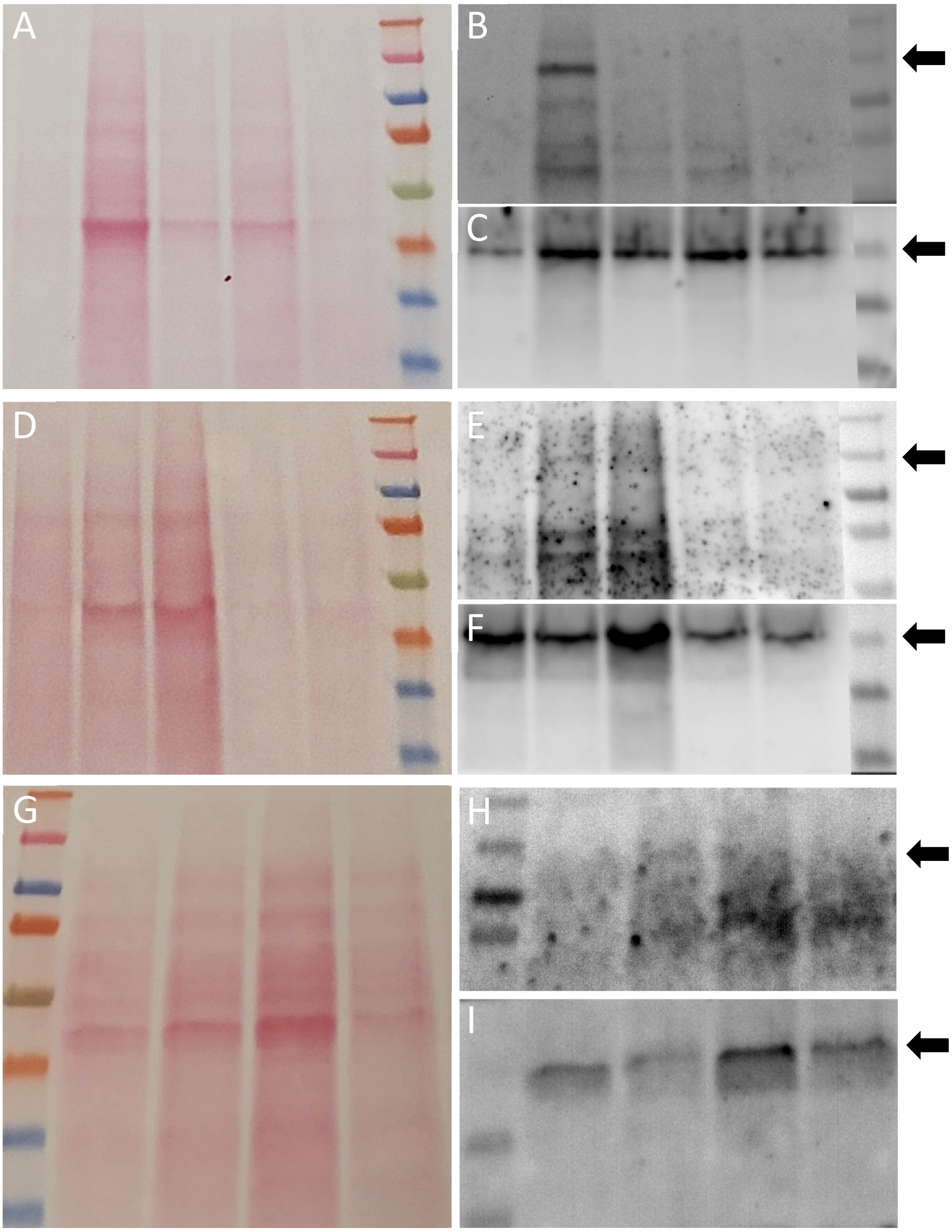

Quantitative analysis of protein levels of iNOS (B,E) and Arg-1 (C,F) was done by Western blot as described in the method part of the manuscript (A-C Blot1, D-F Blot2). 400k non/treated tg microglia cells were taken per condition. Values were normalized to whole protein concentration (A,D). The arrows show the running height of the evaluated bands. The marker used was the Spectra™ Multicolor Broad Range Protein Ladder from Thermo Scientific™ (Catalog number: 26634).
