# Supplementary material for: Parthenolide regulates microglial and astrocyte function in primary cultures from ALS mice and has neuroprotective effects on primary motor neurons
Source: PLoS One. 2025 Mar 18;20(3):e0319866. doi: 10.1371/journal.pone.0319866 (PMC11918366; doi:10.1371/journal.pone.0319866)
Supplement: S1 Table — (DOCX) [file pone.0319866.s008.docx]

**Supplement table 1: Primers used for real time PCR**

| Genes | | Primer sequence (5'->3') (forward/reverse) | Product length |
| --- | --- | --- | --- |
| Vim  (PMID: 28099414) | NM_011701.4 | AGACCAGAGATGGACAGGTGA | 169 |
|  |  | TTGCGCTCCTGAAAAACTGC |  |
| Snap25  (PMID: 28099414) | NM_001291056.1 | AGCAAGGCGAACAACTCGAT | 106 |
|  |  | AGGCCACAGCATTTGCCTAA |  |
| Nefl  (PMID: 28099414) | NM_010910.2 | CAAGGACGAGGTGTCGGAAA | 152 |
|  |  | TGATTGTGTCCTGCATGGCG |  |
| Mbp  (PMID: 28099414) | NM_001025259.2 NM_001025258.2 NM_001025256.2 NM_001025255.2 NM_001025254.2 NM_001025251.2 NM_010777.3 | GAGACCCTCACAGCGATCCAAG | 282 |
|  |  | GGAGGTGGTGTTCGAGGTGTC |  |
| Mog  (PMID: 28099414) | NM_010814.2 | CACCGAAGACTGGCAGGACA | 129 |
|  |  | CCACAGCAAAGAGGCCAATG |  |
| Cx3cr1  (PMID: 36428484) | NM_009987.4 | CTGTTATTTGGGCGACATTG | 93 |
|  |  | AACAGATTTCCCACCAGACC |  |
| Cd11b  (ncbi primer-blast) | NM_001082960.1 NM_008401.2 | GTCTAAGACAGAGACCAAAGTG | 130 |
|  |  | GAAAAAGCCAAGCTTGTATAG |  |
